# Developing Lanthanide-Nitrate Cluster Chemistry toward Rare Earth Separations

**DOI:** 10.1021/acs.inorgchem.5c01730

**Published:** 2025-08-11

**Authors:** Thomas L. McCusker, Alexander Roseborough, Morgan A. McDonald, Sabrina A. Jackson, Frenio A. Redeker, May Nyman, Karah E. Knope

**Affiliations:** † Department of Chemistry, 8368 Georgetown University, 37th and O Streets NW, Washington, District of Columbia 20057, United States; ‡ Department of Chemistry, 153 Gilbert Hall, 2694 Oregon State University, Corvallis, Oregon 97331, United States

## Abstract

Nitrate-decorated hexamers with a [Ln_6_(μ_6_-O)­(μ_3_–OH)_8_]^8+^ core have been reported for nearly every lanthanide ion and are used as precursors for the assembly of functional metal–organic frameworks. Yet, few studies have examined the correlation between the solution and solid-state species, and the formation of mixed-metal clusters. Toward this end, a series of homo- and heterometal lanthanide nitrate hexamers was prepared via pH adjustment of aqueous lanthanide nitrate solutions. Examination of the homometallic europium solutions using Small Angle X-ray Scattering and *n*ESI-MS showed that lower order complexes dominate lanthanide speciation in nitrate media. Yet, powder X-ray diffraction data of the precipitated phase confirmed the formation of [Ln_6_(μ_6_-O)­(μ_3_-OH)_8_(NO_3_)_6_(H_2_O)_12_]·2­(NO_3_)·*n*(H_2_O), **Ln**_**6**_, for Ln = Eu and Tb. For heterometal systems, analysis of the solid-state product by ICP–MS showed the selective incorporation of the heavier rare earths into **Ln**_**6**_. Selectivity was quantified by calculating an average separation factor, which is defined as the ratio of recovery factors of both metals. Further examination of the luminescence behavior of mixed metal [Tb_6–*x*_Eu_*x*_(μ_6_-O)­(μ_3_-OH)_8_(NO_3_)_6_(H_2_O)_12_]·2­(NO_3_)·*n*(H_2_O), with *x* = 1.1–3.6, showed that the relative intensities of the peaks at 489 nm (terbium, ^5^D_4_ → ^7^F_6_) and 690 nm (europium, ^5^D_0_ → ^7^F_4_) trend with the percent incorporation of europium and terbium into the cluster.

## Graphical abstract

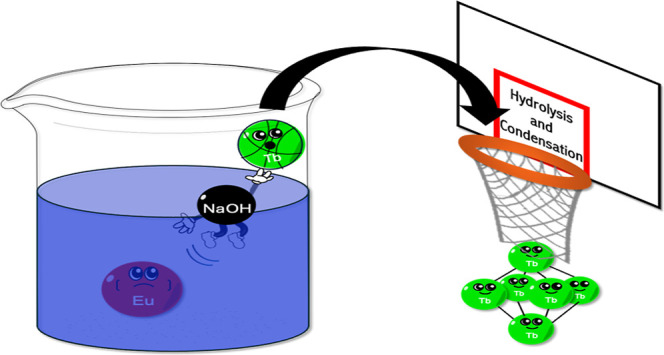

## Introduction

Lanthanide–lanthanide separations remain one of the most difficult on the periodic table due to the chemically coherent nature of the *4f*-elements.^1,2^ The similarity in chemical behavior arises from a common +3 oxidation state and just an 18% decrease in ionic radii from the beginning (La; 1.045 Å) to the end (Lu; 0.861 Å) of the series.^3^ Such subtle changes underpin the chemical coherence of the series and as a result many cases exist where members of the series display identical chemistry within a given system.^3,4^ Despite the difficulty associated with these separations, they remain necessary as the rare earth elements display desirable magnetic and luminescent properties that make them useful in applications ranging from catalysis to optical devices for biomedical imaging.^5–7^

The current paradigm for the separation of these elements is via liquid–liquid extractions from acidic stock solutions (typically either nitrate- or chloride-based systems). Through repeated contact with an organic layer containing an extractant, lanthanides are separated based on slight differences in solubility. This process is inherently atom inefficient—one extractant molecule, such as tri-*n*-butyl phosphate (TBP), extracts one lanthanide atom.^1,8–10^ This process is both time- and energy-intensive and generates tons of acidic waste. Recent work has focused on developing new paradigms for the separation process and has led to a number of alternative separation strategies including the use of lanmodulin, an enzyme capable of selectively binding to rare earth elements (REEs), as well as ion exchange, magnetic, and electrochemical techniques.^11–18^ One promising method that has recently been revisited is selective crystallization of REEs using materials, such as metal–organic frameworks and extended borate networks.^19,20^ While these have been shown to be effective systems for the separation of the lanthanides, the mechanism through which these separations occur is not well understood and, therefore, not widely applicable.

With this in mind, researchers have sought to develop broadly applicable design principles to achieve metal-ion self-sorting. For example, several groups have demonstrated the ability of lanthanide ions to selectively bind not only to organic ligands forming mononuclear complexes, but to supramolecular networks that display cooperative binding and ultimately enhanced selectively for specific metal ions.^21–24^ In a related vein, we now look to establish similar design principles using metal-oxo cluster chemistry. Ln-oxo clusters are a well-established class of materials which typically form via hydrolysis and condensation and the arrested precipitation of -oxo and -hydroxo-bridged oligomers.^25^ The prevalence of these phases for metal ions across the periodic table and the broad conditions under which they form make them an attractive vehicle through which a separation could be achieved. Moreover, exploiting slight differences in hydrolysis behavior can yield distinct cluster chemistries, enabling separations based on the cluster size, solubility, surface ligation, and lability, as demonstrated for Hf/Zr oxocluster systems.^26,27^

In an attempt to further examine the utility of differences in metal ion cluster chemistry in separations, we turned to aqueous Ln systems that are relevant to current REE separation techniques. Of particular interest was the nitrate-decorated lanthanide hexamers, [Ln_6_(μ_6_-O)­(μ_3_-OH)_8_(NO_3_)_6_(H_2_O)_*y*_]·2­(NO_3_)·*n*(H_2_O), **Ln**_**6**_.^28–33^ Importantly, this cluster topology has been reported for almost every lanthanide ion with various amounts of water in the outer-coordination sphere (Table S1). The hexamers are typically synthesized via the slow titration of base into solutions containing dissolved lanthanide nitrate salts. Both the reproducibility of the synthesis and pervasiveness of these phases has allowed them to be used as molecular precursors for the preparation of extended networks.^34^ Yet very few studies describe the correlation between the solution- and solid-state species, or examine mixed-metal systems.^34,35^ Here, we examine the formation of clusters in solution using small-angle X-ray scattering (SAXS) and nanoelectrospray ionization-mass spectrometry (nESI-MS). The chemical (via base adjustment) and electrochemical syntheses of homo- and heterometal Ln_6_-nitrate clusters, including [Ln_6_(μ_6_-O)­(μ_3_-OH)_8_(NO_3_)_6_(H_2_O)_12_]·2­(NO_3_)·*n*(H_2_O), **Ln**_**6**_, for Ln = Eu and Tb, and [Tb_6–*x*_Ln_*x*_(μ_6_-O)­(μ_3_-OH)_8_(NO_3_)_6_(H_2_O)_12_]·2­(NO_3_)·*n*(H_2_O), **Tb**_**6–*x***_ **Ln**_***x***_ (Ln = Pr, Nd, Eu, Sm, Er), are presented. Analysis of mixed-metal samples via inductively coupled plasma–mass spectrometry (ICP–MS) shows that the heavier lanthanide of the pair preferentially incorporates into the solid-state product. For the **Tb**_**6–*x***_ **Eu**_***x***_ series, solid-state luminescence is used to examine the correlation between emission intensity and the relative percentages of lanthanides in the sample. Overall, while the relatively low yield of these phases limits their direct application to a separation technique, evidence for lanthanide self-sorting based on differences in metal ion hydrolysis suggests that cluster-based separations could be broadly applicable to separations of chemically similar metal ions.

## Experimental Methods

### Materials

Lanthanide nitrate hydrates (Ln­(NO_3_)_3_·*n*H_2_O; Ln = Pr, Nd, Sm, Eu, Tb, Er; Strem Chemicals) and 200 proof ethanol (EtOH, Fisher Scientific) were used as received without any further purification. Solid sodium hydroxide pellets (NaOH; Fisher Scientific) were used to make 0.5 M NaOH solutions; pellets were dissolved in nanopure water. Nanopure water (≤0.05 μS)­was purified by a Millipore Direct-Q 3UV water purification system and used in all reactions. ICP–MS standards (SPEX CertiPrep) were diluted in trace metal grade 2% nitric acid (Fisher Scientific) but were otherwise used as purchased. Sodium chloride (NaCl; Fisher Scientific) and sodium nitrate (NaNO_3_; Fisher Scientific) were used as purchased. Other chemicals including glycine (Fisher Scientific), sodium acetate (Fisher Scientific), sodium chloroacetate (Fisher Scientific), and sodium dihydroxybenzoate (Fisher Scientific), were used as received.

### Syntheses

*Base precipitation of **Ln**_**6**_ (Ln = Eu, Tb).* Crystalline powders of homometallic basic lanthanide nitrates of the general formula [Ln_6_(μ_6_-O)­(μ_3_-OH)_8_(NO_3_)_6_(H_2_O)_12_]·2­(NO_3_)·*n*(H_2_O) (**Ln**_**6**_; Ln = Eu and Tb) were prepared by the base adjustment of an aqueous solution, following a method previously reported by Calvez et al. (Figure 1).^32^ The corresponding lanthanide nitrate salt (10 mmol, 4.461 g for Eu and 4.530 g for Tb) was dissolved in a 1:9 solution of H_2_O/EtOH (10 mL) in a 25 mL vial. A solution of 0.5 M sodium hydroxide (1.5 mmol, 3 mL) was then added to the solution dropwise over 10 mins while stirring vigorously. Importantly, the rate of addition seemed to affect the crystallinity of the resulting product. Upon the addition of a base, the solution immediately became cloudy, consistent with the precipitation of the desired phase. The precipitate was isolated using vacuum filtration, and the solid was dried under ambient conditions overnight. The effect of different bases, including lithium hydroxide and triethylamine, was investigated. Different alkali metal hydroxides had no apparent effect on the resulting phase, while triethylamine led to the immediate precipitation of a non-crystalline phase thought to be lanthanide hydroxide.

**Figure 1:**
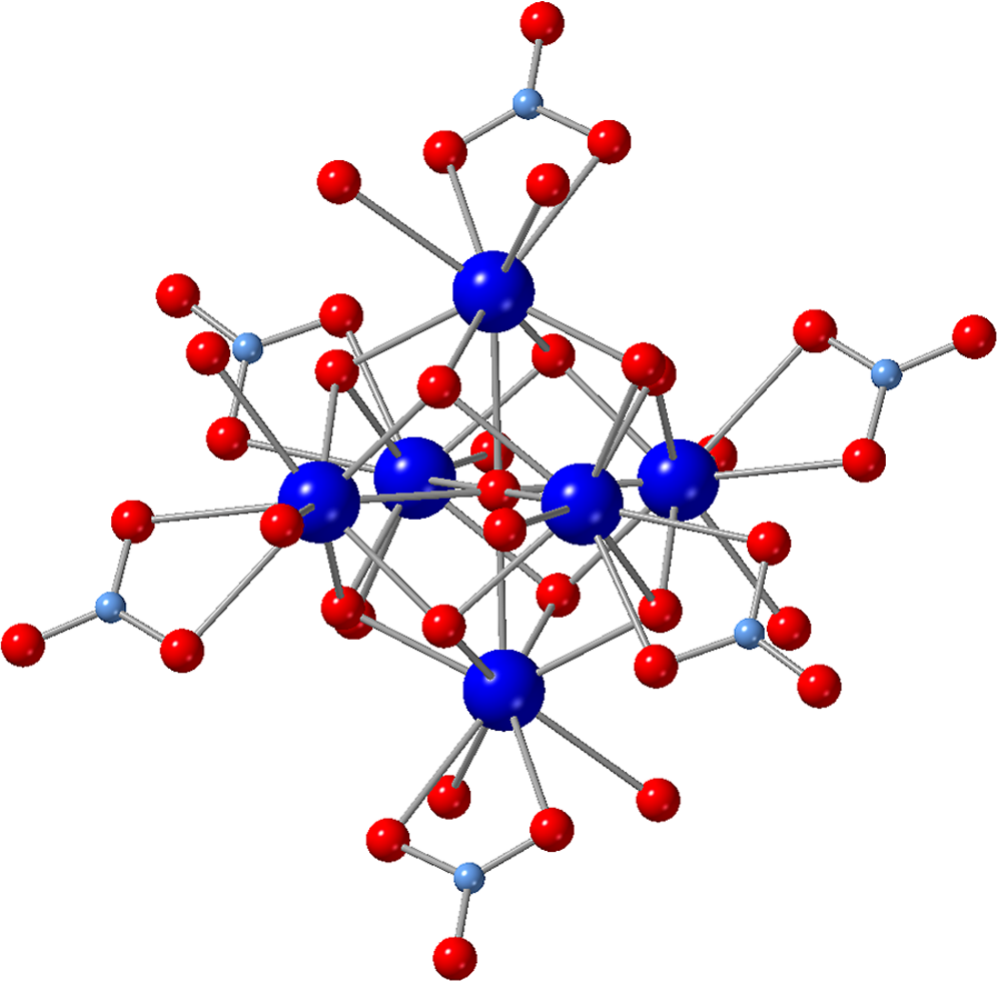
Ball-and-stick illustration of **Ln**_**6**_, [Ln_6_(μ_6_-O)­(μ_3_-OH)_8_(NO_3_)_6_(H_2_O)_12_]^2+^, which was rendered using ICSD 418810. Lanthanide atoms are depicted in blue, oxygen in red, and nitrogen in light blue. All hydrogens have been omitted for clarity.

Heterometallic phases, **Tb**_**6–*x***_ **Ln**_***x***_ (Ln = Pr, Nd, Eu, Sm, Er), were prepared following the same synthetic approach. The corresponding lanthanide nitrate salts (10 mmol total lanthanide) were dissolved in a 1:9 solution of H_2_O/EtOH (10 mL) in a 25 mL vial. A solution of 0.5 M sodium hydroxide (1.5 mmol, 3 mL) was then added dropwise to yield the desired product. For **Tb**_**6–*x***_ **Eu**_***x***_, a series of mixed metal reactions with Tb/Eu ratios of 25:75, 43:57, 66:33, and 75:25 mol % were examined. For all other **Tb**_**6–*x***_ **Ln**_***x***_ (Ln = Pr, Nd, Sm, Er), the Tb/Ln ratios were maintained at 50:50. Powder X-ray diffraction (PXRD), inductively coupled plasma–mass spectrometry (ICP–MS), and luminescence measurements were collected on these phases.

*Electrochemical Preparation of* ***Eu***_**6**_. As noted previously, addition of base to the lanthanide nitrate solutions led to the immediate precipitation of the targeted phases. This limited our ability to study solution speciation. Thus, to avoid rapid precipitation, solutions were coulometrically titrated. Importantly, Eu was chosen for these studies, as it has an easily identifiable isotopic fingerprint and thus is well suited for solution studies using nano-electrospray ionization mass spectrometry (*n*ESI-MS). Aqueous solutions of europium nitrate were prepared via dissolution of Eu­(NO_3_)_3_·6­(H_2_O) (10 mmol, 4.461 g) in H_2_O (8 mL). The solution (10 mmol of Eu; 8 mL) was then placed in one compartment of a two-component electrochemical cell. A solution of sodium chloride dissolved in H_2_O (1 M) was placed in the other compartment. A platinum wire was used for the electrodes, with the working electrode placed in the Eu solution and the counter electrode in the NaCl solution. The solution with the working electrode was stirred over the course of the experiment. A current of −73 to −74 mA was then applied to the system using a Voltalab Radiometer PGZ 402 Universal Pulse Dynamic-EIS voltameter. For solution studies, the titration was stopped prior to the precipitation of **Eu**_**6**_; however, prolonged titrations were performed to confirm the formation of **Eu**_**6**_ from these solutions. Small-angle X-ray scattering (SAXS) and nano-electrospray ionization-mass spectrometry (*n*ESI-MS) were completed on these solutions.

For europium, various synthetic parameters were explored in an effort to bias the solution species toward hexameric units: lanthanide concentrations were varied from 50 to 625 mM, and sodium nitrate (0 to 100 mM) and/or organic ligands (100 mM of glycine, acetate, chloroacetate, or dihydroxybenzoate) were added to the reaction solutions. On average, the pH of the solutions increased from 4.9 to 6.2, which was similar to the change in pH observed in the chemical titration.

### Phase Identification via Powder X-ray Diffraction (PXRD)

Powder X-ray Diffraction patterns were collected on a Rigaku Ultima IV diffractometer (Cu Kα = 1.524 Å) for phase identification (Figures S1–S3). Powdered samples were placed on zero-background holders. Data were collected from 3° to 40° 2θ with a 1° per minute scan speed, 0.02° step size, and 2/3 ° divergence slit. Simulated PXRD patterns were generated from published crystal structures (ICSD# 418810, ICSD# 418815, and ICSD# 170885) and compared to experimental data (Figures S1–S3).

### Small Angle X-ray Scattering (SAXS)

Small-angle X-ray scattering data were collected on an Anton Paar SAXSess instrument (Cu Kα = 1.54 Å) with line collimation. A 2-D image plate was used for data collection in the *q* = 0.018–2.5 Å^–1^ range with the lower q range limited by the beam attenuator. Scattering data of neat water was collected for background subtraction. Scattered samples included lanthanide nitrate salts dissolved in water both pre- and postelectrolysis (Figure 2). These samples were filtered using a 0.45 μm membrane filter and sealed in 1.5 mm glass capillaries (Hampton Research) for measurements. Scattering data were collected for 30 min. SAXSQUANT software was used for data collection and processing (normalization, primary beam removal, background subtraction, desmearing, and smoothing to remove extra noise created by the desmearing routine). Data analysis was done in IgorPro using IRENA macros.^36^ SolX was used to simulate scattering data for Eu monomer and hexamer units based on modified structure files of the previously reported Eu hexamer.^29,37^

**Figure 2:**
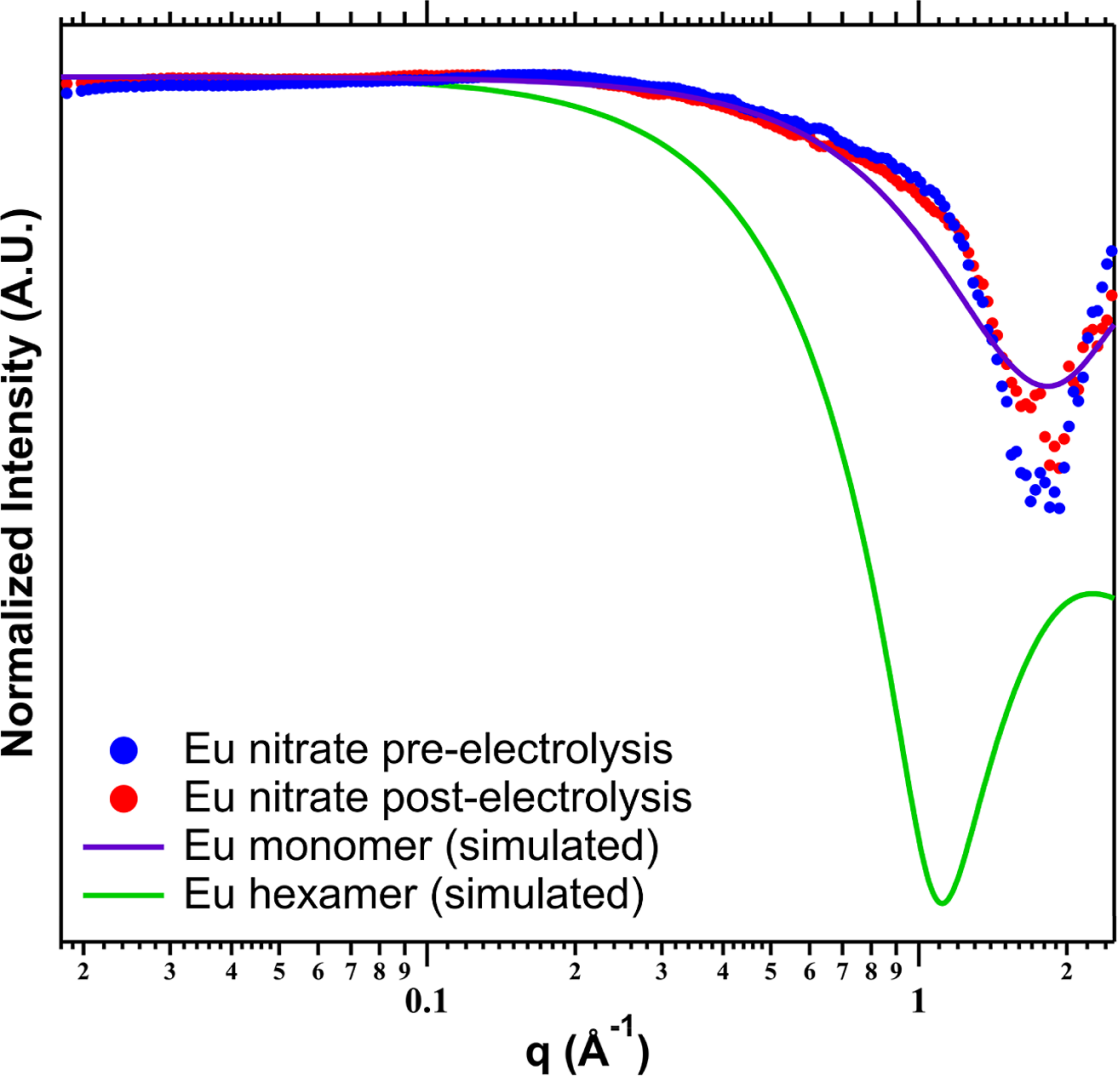
SAXS curves of europium-nitrate solution pre- (blue) and post- (red) electrolysis along with simulated SAXS curves for a nitrate-decorated Eu monomer (purple) and hexamer (green). Comparison of the experimental and simulated patterns suggests that monomeric units are the dominant solution species.

### Nano-Electrospray Ionization-Mass Spectrometry (*n*ESI-MS)

*n*ESI-MS spectra were acquired from *m*/*z* 50–3000 using a SciEx QStar XL quadrupole time-of-flight mass spectrometer. Nanospray emitters (3–5 μm ID) were made from borosilicate glass capillaries (World Precision Instruments, 1 mm OD, 0.75 mm ID) by using a Sutter Instrument Co. Model P-87 Flaming/Brown micropipette puller. Europium nitrate solutions from pre-electrolysis, post-electrolysis, and post-titration stages were chosen for analysis due to the easily identifiable isotopic fingerprint of Eu (Figure S4). These solutions were diluted to ∼1.2 mM and loaded into the emitters. Mass spectra were obtained by applying 1700 V through a platinum wire with a plate voltage of 500 V. Molecular formulas within a mass accuracy of 100 ppm were considered for assignments (see Table S2 for experimental and theoretical *m*/*z*-values of individual peak assignments). Peak assignments were validated by a visual comparison of experimental isotopic patterns with theoretical patterns simulated in R using the enviPat package.^38,39^

### Inductively Coupled Plasma-Mass Spectrometry (ICP–MS)

ICP–MS data were collected using an Agilent 7800 series ICP quadrupole with argon plasma. Standards of lanthanide nitrates were prepared at 100, 75, 50, 25, 10, and 5 ppb in trace metal grade 2% nitric acid and used to construct calibration curves (Figure S5). Approximately 2 mg samples of **Tb**_6–*x*_ **Ln**_***x***_ (Ln = Pr, Nd, Eu, Sm, Er) were dissolved in trace metal grade 2% nitric acid. The relative mol % of each Ln in the heterometallic phases was then determined by comparison of the values obtained from these measurements with the appropriate calibration curves (Tables S3–S5).

### Luminescence of Homometal Ln_6_ (Ln = Eu, Tb) and Heterometal Tb_6–*x*_Eu_*x*_ Phases

Excitation and emission spectra for homometallic phases, **Eu**_**6**_ and **Tb**_**6**_, were collected on a Horiba PTI QM-400 spectrofluorometer (Figures S6, S7). Solid-samples were placed between two quartz slides, and spectra were collected at room temperature using 5 nm slit widths. Long pass filters were used to limit harmonic peaks from the lamp. Once it was confirmed that excitation at 365 nm yielded both Eu and Tb emission, emission spectra for **Eu**_**6**_ and **Tb**_**6**_ as well as the heterometallic series, **Tb**_**6–*x***_ **Eu**_***x***_ (*x* = 1.1–3.6) were collected using a Craic 508 PV microspectrophotometer attached to a Zeiss Axioscope 5 microscope. Spectra obtained from the CRAIC 508 PV microspectrometer exhibited a more consistent background intensity and thus allowed for a better comparison of the relative intensities of the Eu- and Tb-based emission across the **Tb**_**6–*x***_ **Eu**_***x***_ (*x* = 1.1–3.6) series.

## Results and Discussion

### Synthetic Considerations

In this work, lanthanide-nitrate hexamers of composition [Ln_6_(μ_6_-O)­(μ_3_-OH)_8_(NO_3_)_6_(H_2_O)_12_]·2­(NO_3_)·*n*(H_2_O) (Ln = Eu, Tb) and [Tb_6–*x*_Ln_*x*_(μ_6_-O)­(μ_3_-OH)_8_(NO_3_)_6_(H_2_O)_12_]·2­(NO_3_)·n­(H_2_O) (Ln = Pr, Nd, Eu, Sm, Er) were prepared by chemical titration.^32^ Phase identification was based on powder X-ray diffraction (Figures S1–S3), with the experimental powder patterns showing good agreement with the patterns calculated for the dihydrate (ICSD 170885) and trihydrate (ICSD 418810) of [Ln_6_(μ_6_-O)­(μ_3_-OH)_8_(NO_3_)_6_(H_2_O)_12_]·2­(NO_3_). Importantly, both compounds exhibit the same cluster core and ligand decoration, and differ only in the number of outer sphere water molecules. As shown in Figure 1, the previously reported cluster features six Ln metal centers bridged by eight μ_3_-hydroxo groups and a central μ_6_-oxo. The cluster core is decorated by six nitrate groups and twelve water molecules yielding a dicationic cluster. The cationic cluster is then charge balanced by two outer-coordination sphere nitrates. The slow addition of a sodium hydroxide solution to a lanthanide-nitrate stock solution resulted in immediate precipitation of the targeted phases, with the pH of the resulting solution being approximately 6.5–7. The crystallinity of the precipitate ranged significantly as variations in scale, stirring, or isolation via filtration led to pronounced losses in crystallinity. Yields (based on lanthanide) ranged from 1%–20%. While the targeted phases could be readily prepared using this synthetic methodology, there were two main shortcomings of this approach: the solid-state product only accounted for a fraction of the total lanthanide in the reaction, and it was unclear whether immediate precipitation of the hexamer precluded its detection in solution. It is worth noting; however, that previous work by Zak et al. and Marsh et al. has shown these hexameric units to have limited solution stability.^28,40^ Nonetheless, to limit local pH gradients that form upon base addition and avoid rapid precipitate formation, an electrochemical approach was employed, with the electrolysis of water yielding OH^–^ and more uniform increase in pH. Solutions were coulometrically titrated to pH ∼6.2 over 2 min, with additional time resulting in the crystallization of **Eu**_**6**_ on the electrode surface. Both pre- and postelectrolysis solutions were examined using SAXS (Figure 2) and *n*ESI-MS (Figures 3, 4) to assess the prevalence of the hexameric unit in the solution phase.

**Figure 3:**
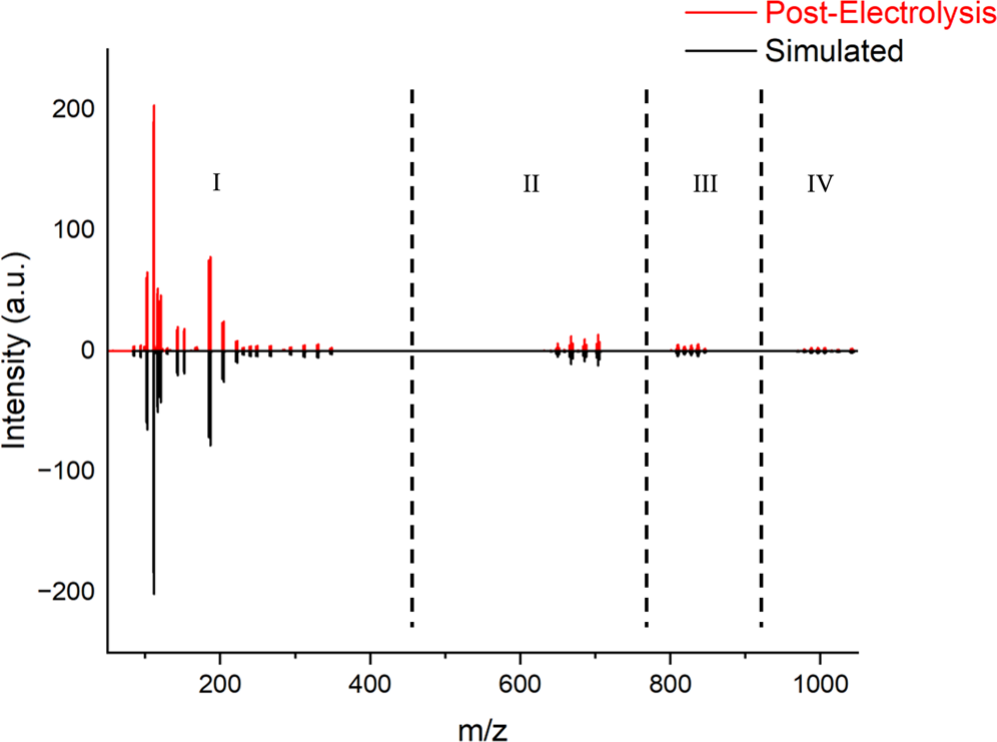
*n*ESI-MS spectrum of a Eu post-electrolysis solution with the (I) monomer region, (II) dimer/tetramer, (III) pentamer, and (IV) trimer/hexamer regions labeled.

**Figure 4:**
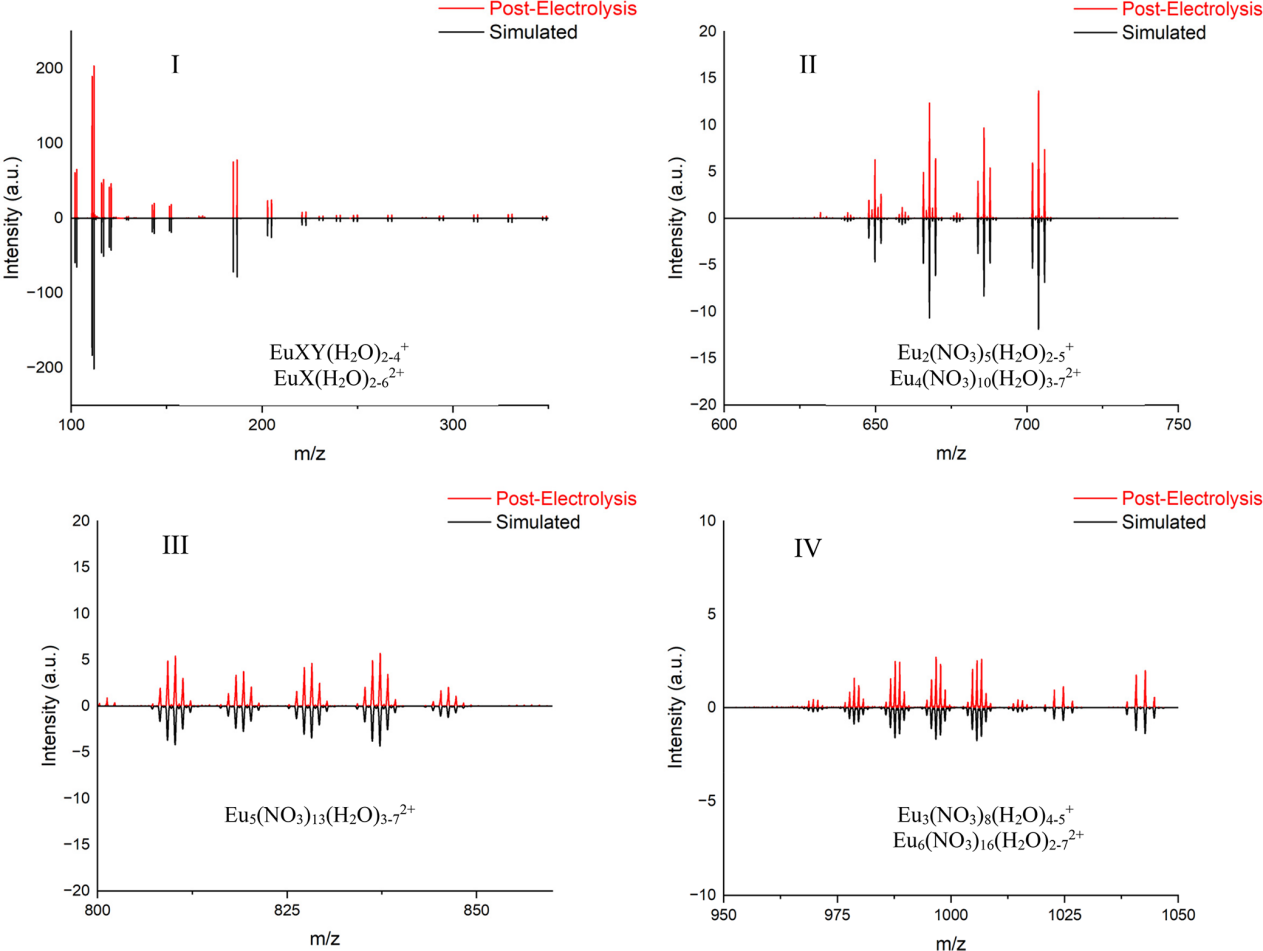
*n*ESI-MS spectrum of a Eu post-electrolysis solution with individual regions (I–IV) highlighted. X, Y = OH, NO_3_.

### Solution Speciation

In an effort to determine the prevalence of the hexameric units in solution, Small-Angle X-ray Scattering (SAXS) and nano-electrospray ionization mass spectrometry (*n*ESI-MS) data were collected on the Eu nitrate solutions both pre- and post-electrolysis. The SAXS curves, along with simulated profiles for monomeric and hexameric units, are shown in Figure 2. The slope of the scattering curve for the solutions over *q* = 0.1–1 Å^–1^ matches closely with a simulated monomeric unit, indicating that despite the precipitation of the hexameric unit, oligomeric clusters are not the dominant solution species. Previous studies have shown that **Ln**_**6**_ compounds display low solubility in water,^28,40^ and taken together with the current work, this may indicate that lanthanide hexamers are not the thermodynamically favored product of Ln­(III) ions under these conditions.

Nano-electrospray ionization-mass spectrometry (*n*ESI-MS) was employed (Figure 3) in order to further probe solution speciation and validate the findings from SAXS. Like the SAXS data, mass spectra of pre- and post-electrolysis solutions were nearly identical, indicating that the solution speciation in these systems is remarkably similar. Additionally, the *n*ESI-MS of the postchemical titration solution was nearly identical to that of the pre- and post-electrolysis solutions (Figure S4), suggesting similar solution species. Both the pre-titration and post-titration spectra feature four distinct regions (Figures 3 and 4): (I) monomeric species (*m*/*z* 100–350), (II) dimeric and tetrameric species (*m*/*z* 630–710), (III) pentameric species (*m*/*z* 800–850), and (IV) trimeric and hexameric species (*m*/*z* 950–1050). Peak intensities are the highest in the monomeric region and rapidly decrease with higher order oligomers. The peaks in the monomeric region could be assigned to doubly charged europium hydroxides and nitrates EuX­(H_2_O)_*n*_^2+^ (X = OH, NO_3_; *n* = 2–6; *m*/*z* = 100–170) and singly charged hydroxides and nitrates EuXY­(H_2_O)_*n*_^+^ (X, Y = OH, NO_3_; *n* = 0–4, *m*/*z* = 180–350). Peaks in region II may be assigned to Eu_2_(NO_3_)_5_(H_2_O)_*n*_^+^ (*n* = 2–5) and Eu_4_(NO_3_)_10_(H_2_O)_*n*_^2+^ (*n* = 3–7). The doubly charged pentamers in region III were identified as Eu_5_(NO_3_)_13_(H_2_O)_*n*_^2+^ (*n* = 3–7) and the peaks in region IV were consistent with the isotopic patterns of Eu_3_(NO_3_)_8_(H_2_O)_*n*_^+^ (*n* = 4, 5) and Eu_6_(NO_3_)_16_(H_2_O)_*n*_^2+^ (*n* = 2–7). The very low intensity of hexameric species compared to monomeric species could be partly due to the instrument bias toward lower *m*/*z* species; however, given the SAXS data, these findings suggest that the majority of the lanthanide ions in solution exist as monomeric units. It should also be mentioned that the hexameric unit formulated from the *n*ESI-MS spectra is not consistent with the precipitated phase. In fact, no hydroxide species were observed, apart from those in the monomeric region. It is likely that these species could be interpreted as precursors to the final hydrolysis products that are observed in the solid state.

The observation of other oligomeric species in addition to hexamers was somewhat surprising when considering the lack of diversity in the solid state. This, along with the predominance of the monomeric complexes over the hexanuclear clusters, highlights a stark disparity in the solution and solid-state species, with the latter exclusively exhibiting the hexamer. While the structural diversity of this system in solution is notable, the lack of solution stability of the hexameric cluster may admittedly limit the possibility of applying this system to a liquid–liquid separation.

### Evidence for Lanthanide Selectivity in the Precipitated Phase

Despite relatively low yields and evidence that hexameric units are not prevalent in solution, a series of mixed metal **Tb_6–*x*_Eu_*x*_** and **Tb** **_6–*x*_Ln** **_*x*_** (Ln = Pr, Nd, Sm, Er) compounds were prepared to assess any preferences for lanthanide incorporation into the precipitated phase. Inductively Coupled Plasma-Mass Spectrometry (ICP–MS) data was collected on mixed metal precipitates dissolved in 2% HNO_3_. Lanthanide incorporation was determined by comparing the data to calibration curves, which gave the relative percentages of each lanthanide in the solid-state reaction product. Invariably, the ratio of heavier lanthanide to lighter lanthanide was higher than the ratios of the lanthanides added to the system. For the **Tb**_**6–*x***_ **Eu**_***x***_ series, reactions with stoichiometric ratios of roughly 25:75, 43:57, 66:33, and 75:25 were examined. As shown in Figure 5, the mol % of europium added to the reaction mixture is consistently lower than that observed in the solid-state product. Only at initial ratios of 25:75 Tb/Eu does the hexamer incorporate more Eu than Tb. Interestingly, the difference in mol % for the lanthanides in these systems can be correlated with differences in the charge density of the metal ions. The ICP–MS results for Tb_6–*x*_Ln_*x*_ (Ln = Pr, Nd, Sm, Er) that precipitate from 50:50 Tb/Ln solutions are shown in Figure 6. Notably, there is a sharp decrease in the mole % of the lighter lanthanide in the precipitated Tb_6–*x*_Ln_*x*_ from Sm to Pr. This trend is consistent with the difference in ionic radii between Tb and the second metal, with a contraction of 3.6% from Sm to Tb and a 7.6% contraction from Pr to Tb. In fact, the 50:50 Pr/Tb solution yields a hexameric phase that is over 90% terbium (Figure 6). Furthermore, inclusion of a heavier lanthanide as compared to Tb (i.e, Tb/Er) yields a solid-state product that is relatively low in Tb (less than 40%) as compared to the initial concentrations.

**Figure 5:**
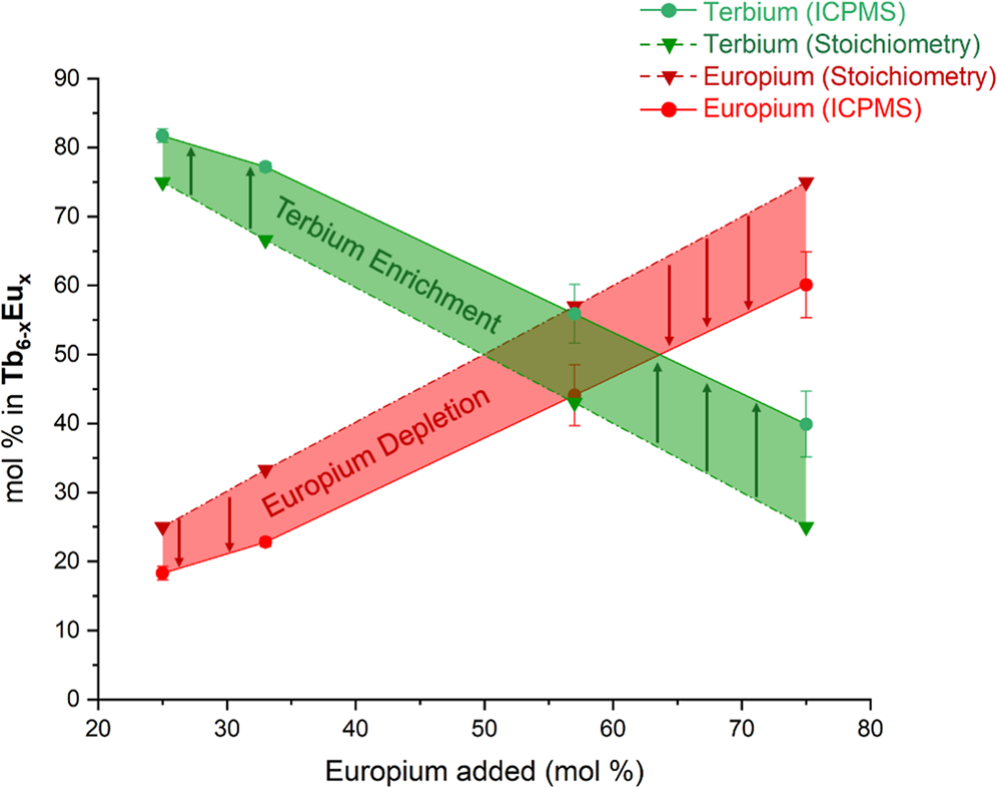
Amount of terbium and europium in each **Tb**_**6–*x***_ **Eu**_***x***_ hexamer compared to the expected amount as a function of mol % of europium added to the reaction.

**Figure 6:**
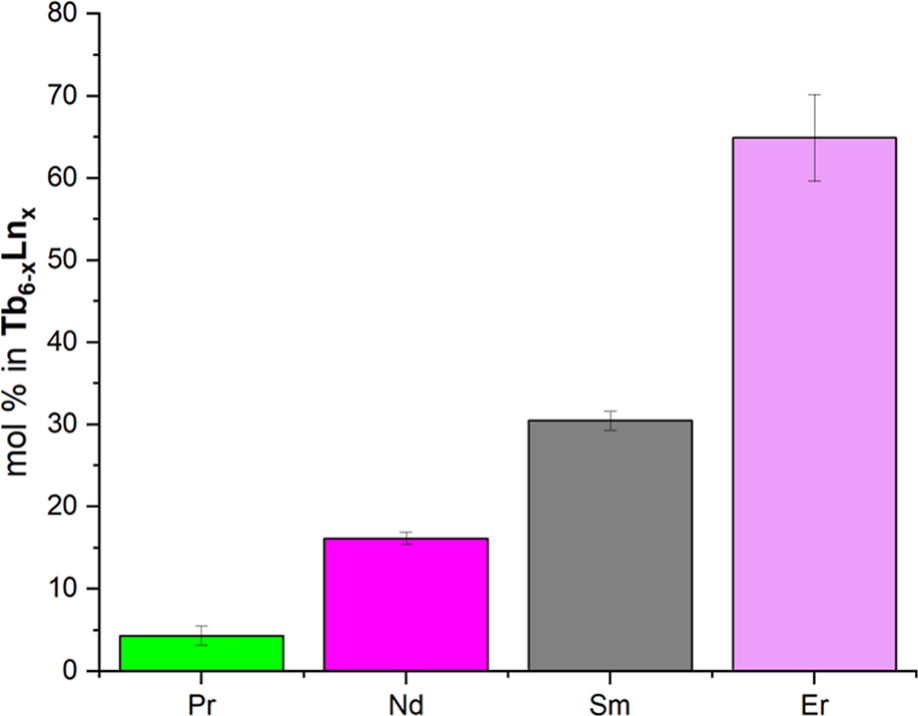
Amount of REE in **Tb**_**6–*x***_ **Ln**_***x***_ hexamers precipitated from 1:1 mixtures of Ln and Tb.

Taken together, these results may imply that hexamer formation (and precipitation) is more favorable for the heavier, more charge dense lanthanides, which preferentially incorporate into the solid-state product. Such a trend is consistent with the hydrolysis constants of the lanthanide ions (p*K*_h1_) which are defined in eq 1.

1 $${\mathrm{Ln}}^{3+}+H_{2}O⇄\mathrm{Ln}{\left(OH\right)}^{2+}+H^{+}\;\left(pK_{h1}\right)$$

The hydrolysis constants reflect the increasing Brønsted acidity of the ions, with p*K*_h1_ decreasing across the series (p*K*_h1_ Pr: 8.32 ± 0.04, Nd: 8.24 ± 0.07, Sm: 8.02 ± 0.02, Eu: 7.91 ± 0.05, Tb: 7.74 ± 0.04, and Er: 7.63 ± 0.02).^41^ Further, these results suggest that differences in hydrolysis and condensation behavior across the series may be leveraged in separations to selectively precipitate heavier REEs. In fact, separation factors (*S*_Tb/Ln_) were calculated based on a previously reported method using eq 2.^42^

2 $$S_{Tb/\mathrm{Ln}}=\frac{mol_{final\;Tb}}{mol_{final\;\mathrm{Ln}}}/\frac{mol_{initial\;Tb}}{mol_{initial\;\mathrm{Ln}}}$$

For the **Tb**_**6–*x***_ **Ln**_***x***_ (Ln = Pr, Nd, Sm, Er) series (Tables S6, S7), selectivity was found to increase with increasing differences in charge density between Tb and the other Ln ion. This trend is highlighted in Figure 7, which shows the log of the separation factor for each element in the **Tb**_**6–*x***_ **Ln**_***x***_ series plotted as a function of p*K*_h1_ of the “second” lanthanide ion.

**Figure 7:**
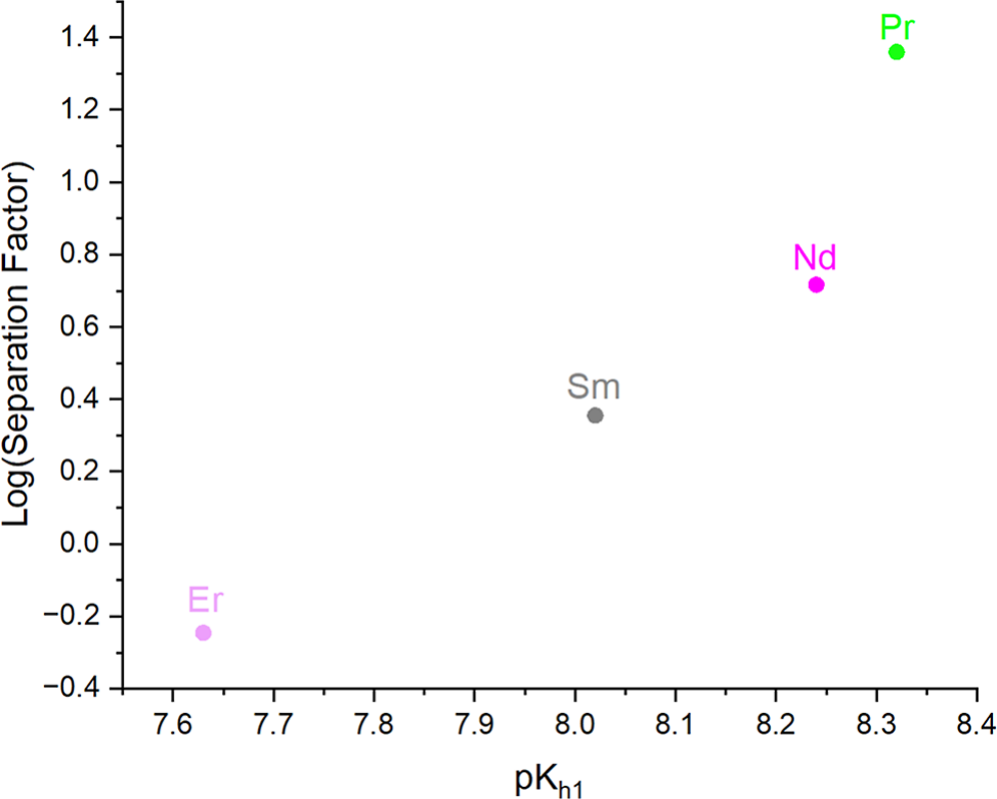
Log of the separation factors for **Tb**_**6–*x***_ **Ln**_***x***_ as a function of p*K*_h1_ displaying the relationship between the propensity of the metal ion to hydrolyze and its precipitation in a mixed metal system.

### Luminescence as a Handle for Understanding Tb/Eu Ratios

The luminescence behavior of the **Tb**_**6–*x***_ **Eu**_***x***_ series was examined to develop an analytical handle for understanding the relative incorporation of these lanthanides in the solid state. Emission spectra were collected with an excitation wavelength of 365 nm, and for both Eu and Tb, peaks characteristic of *f–f* transitions were observed (Figure 8).^43,44^ Invariably, the spectra exhibited europium signatures at 580 nm (^5^D_0_ → ^7^F_0_), 592 nm (^5^D_0_ → ^7^F_1_), 617 nm (^5^D_0_ → ^7^F_2_), 650 nm (^5^D_0_ → ^7^F_3_), 690 nm (^5^D_0_ → ^7^F_4_), and 696 nm (^5^D_0_ → ^7^F_4_) as well as peaks characteristic of terbium at 489 nm (^5^D_4_ → ^7^F_6_), 546 nm (^5^D_4_ → ^7^F_5_), and 620 nm (^5^D_4_ → ^7^F_3_). As shown in Figure 8, the intensity of the peaks varied as expected based on the relative ratios of Eu and Tb in the solid phase, with the emission color spanning from bright red (100% Eu) to orange, yellow, and finally green (100% Tb). CIE coordinates of the homo- and heterometal compounds are provided in Figure S8. Interestingly, the peaks at 489 nm (terbium, ^5^D_4_ → ^7^F_6_) and 690 nm (europium, ^5^D_0_ → ^7^F_4_) trend nicely with the relative ratios of Tb and Eu in the reaction product (Figure 8). Interestingly, similar quenching for terbium peaks with an increasing europium content has been observed in other Eu/Tb mixed-metal systems.^44^ While the intensity ratio between the 489 and 690 nm peaks appears to be a possible diagnostic handle for understanding the europium/terbium content within the solid-state, further investigation into the luminescence pathway is warranted in order to fully understand the basis of this trend in the peak intensity.

**Figure 8:**
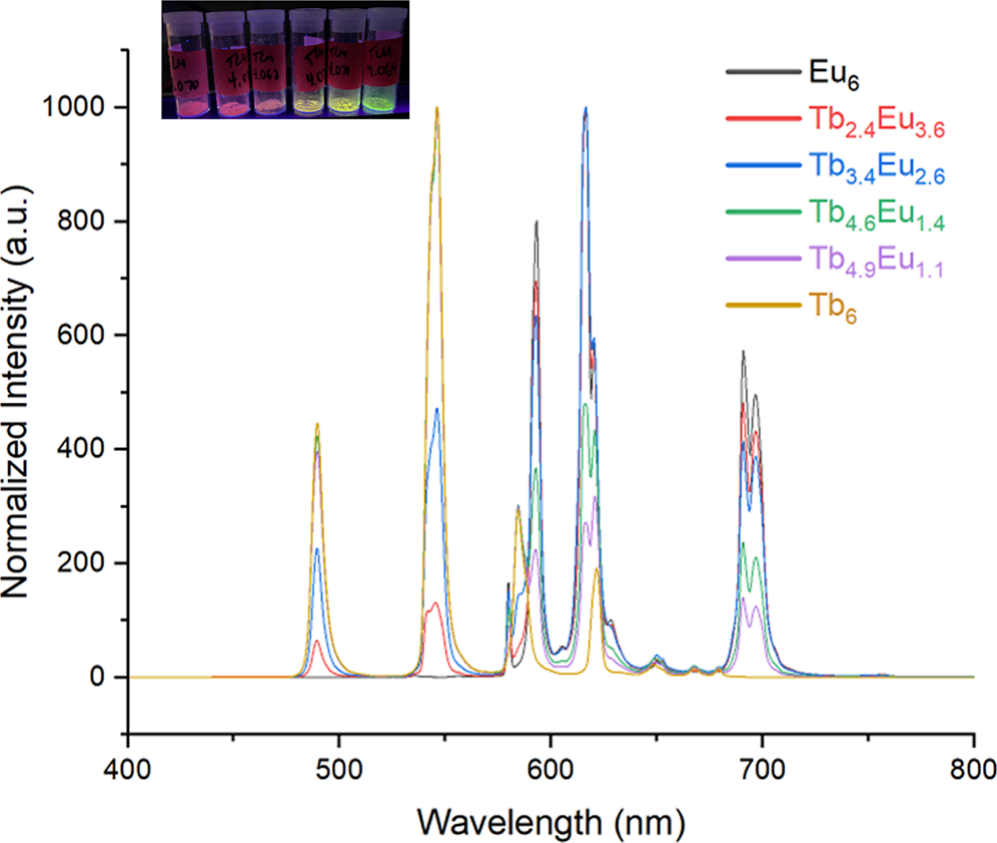
Emission spectra of **Tb**_**6–*x***_ **Eu**_***x***_. All spectra were collected with an excitation wavelength of 365 nm.

## Conclusions

In this work, homo- and heterometal lanthanide hexamers of composition [Ln_6_(μ_6_-O)­(μ_3_-OH)_8_(NO_3_)_6_(H_2_O)_12_]·2­(NO_3_)·*n*(H_2_O) (Ln = Eu, Tb) and [Tb_6–*x*_Ln_*x*_(μ_6_-O)­(μ_3_-OH)_8_(NO_3_)_6_(H_2_O)_12_]·2­(NO_3_)·*n*(H_2_O) (Ln = Pr, Nd, Eu, Sm, Er) were prepared by chemical and electrochemical titration. Importantly, while the hexameric species could readily be precipitated from aqueous nitrate solutions, such species were not observed in solution using *n*ESI-MS nor SAXS; lower order complexes (i.e., monomers) appeared to be the dominant solution species. Nonetheless, the examination of the precipitated phases using ICP–MS showed that for heterometal clusters, heavier lanthanides selectively constituted the solid-state reaction products. Such observations are consistent with the hydrolysis and condensation behavior of the lanthanide ions, with the lanthanide contraction yielding a systematic decrease in the ionic radius and a concurrent increase in the charge density across the series. Additionally, for luminescent lanthanide ions, the emission profiles could provide a useful handle for determining cluster composition, with the ratio of the terbium, ^5^D_4_ → ^7^F_6_ (489 nm) and the europium, ^5^D_0_ → ^7^F_4_ (690 nm) peaks trending with the mol % of the Ln ions in the solid phase. While this system is promising due to the self-sorting behavior of the lanthanide ions that may be attributed to differences in hydrolysis and condensation, the absence of hexanuclear clusters in the solution state as determined by SAXS and *n*ESI-MS data admittedly limits its applicability. Nonetheless, the data suggest that metal-oxo clusters may hold potential in lanthanide separations, and as such, other cluster-based systems warrant consideration.

## Supplementary Material



## Notes

Summary of reported Ln nitrate hexamers, powder X-ray diffraction patterns of homo- and heterometallic phases, *n*ESI-MS, representative ICP–MS, separation factors calculated for **Tb_6–*x*_Ln_*x*_**, excitation and emission spectra for **Eu**_**6**_ and **Tb**_**6**_, and CIE coordinates for **Tb**_**6–*x***_ **Eu**_***x***_ series (PDF)
