# Supplementary material for: Developing Lanthanide-Nitrate Cluster Chemistry toward Rare Earth Separations
Source: Inorg Chem. 2025 Aug 11;64(33):16789–97. doi: 10.1021/acs.inorgchem.5c01730 (PMC12381851; doi:10.1021/acs.inorgchem.5c01730)
Supplement: Supplementary file 1 [file ic5c01730_si_001.pdf]

SUPPORTING INFORMATION

DEVELOPING LANTHANIDE-NITRATE CLUSTER CHEMISTRY TOWARD RARE EARTH SEPARATIONS

Thomas L. McCusker,[a] *Alexander Roseborough,*[b] *Morgan A. McDonald,*[a] *Sabrina A. Jackson,*[a] *Frenio A. Redeker,*[a] *May Nyman,*[b] *and Karah E. Knope*[a,*]

[a] Department of Chemistry, Georgetown University, 37th and O Streets NW, Washington, D.C., 20057, United States
[b] Department of Chemistry, Oregon State University, Corvallis OR, 97331 USA

*kek44@georgetown.edu

Table of Contents

1. **Summary of Reported Lanthanide Nitrate Hexamers** S2
2. **Powder X-ray Diffraction Patterns for Homo- and Heterometallic Phases** S3
3. **Nano-Electrospray Ionization-Mass Spectrometry** S6
4. **Representative ICP-MS Data** S8
5. **Separation Factors for $Tb_{6-x}Eu_x$** S11
6. **Luminescence Data of $Tb_{6-x}Eu_x$** S12
7. **References** S14

1. Summary of Reported Lanthanide Nitrate Hexamers

Table S1: A list of reported nitrate decorated lanthanide hexamers.

Formula	DOI	CSD Numbers	ref
$[Ln_6(\mu_6{-}O)(\mu_3{-}OH)_8(H_2O)_{12}(\eta^2{-}NO_3)_6](NO_3)_2{\cdot}xH_2O$ (Ln=Y, Sm, Eu, Gd, Tb, Dy, Ho, Er, Tm, Yb, Lu; *x*=3, 4, 5, 6)	10.1016/j.jallcom.2009.03.118	418808-418844	1
$[Ln_6(\mu_6\text{-}O)(\mu_3\text{-}OH)_8(NO_3)_6(H_2O)_{12}](NO_3)_2{\cdot}xH_2O$ (Ln = Gd, Tb, Dy, Ho, Er, Y, and Yb) (x= 2)	10.1021/ic0401086	414225, 414323, 414224, 414326, and 414318	2
$[Ln_{a6x}Ln_{b6-6x}O(OH)_8(NO_3)_6]^{2+}$ (Ln_a= Y or Tb, Ln_b= Tb or Eu)	10.1021/ic4008697	N/A	3
$[Ln_6O(OH)_8(NO_3)_6(H_2O)_n]^{2+}$ (n = 12 for Ln = Sm–Lu and Y, n = 14 for Ln = Pr and Nd)	10.1021/acs.inorgchem.5b00947	N/A	4
$Ln_6(\mu_6\text{-}O)(\mu_3\text{-}OH)_8(NO_3)_6(H_2O)_{14}]{\cdot}2NO_3{\cdot}2H_2O$ (Ln = Pr or Nd)	10.1016/j.ica.2007.12.008	1731582	5
$[Ln_6(\mu_6\text{-}O)(\mu_3\text{-}OH)_8(NO_3)_6(H_2O)_{12}]{\cdot}2NO_3{\cdot}2H_2O$ (Ln = Sm–Lu and Y)	10.1021/acs.inorgchem.9b02668	N/A	6
$[Ln_6(O)(OH)_8(H_2O)_{12}(NO_3)_6](NO_3)_2$ (Ln=Y, Gd, Er, and Ho)	10.1039/C6DT03733J	N/A	7
$[Ln_6O(OH)_8(NO_3)_6(H_2O)_x]{\cdot}2NO_3{\cdot}2H_2O$ (x=12,14, or 16) (Ln = Ce–Lu (except Pm) or Y)	10.1016/j.crci.2010.03.029	1733383	8
$[Ln_6O(OH)_8(H_2O)_{12}(NO_3)_6](NO_3)_2{\cdot}xH_2O$, (Ln=Sm, Dy, Er; *x*(Sm)=6, *x*(Dy)=5, *x*(Er)=4)	10.1016/S0925-8388(97)00015-7	1644028-1644030	9
$[Nd_6O(OH)_8(H_2O)_{14}(NO_3)_6](NO_3)_2{\cdot}2H_2O$	10.1134/S1063774507020101	N/A	10
$[Dy_6(\mu_6\text{-}O)(\mu_3\text{-}OH)_8(H_2O)_{12}(NO_3)_6](NO_3)_2(H_2O)_2$	10.1039/D0MA00648C	1571865	11
$[Ln_6(\mu_6\text{-}O)(\mu_3\text{-}OH)_8(H_2O)_{12}(NO_3)_6](NO_3)_2{\cdot}xH_2O$ (Ln=Y, Gd, Yb, x(Y, Yb)=4, x(Gd)=5)	10.1016/0925-8388(94)90795-1	1635689-1635691	12

2. Powder X-ray Diffraction Patterns for Homo- and Heterometallic Phases

Powder X-ray diffraction data were collected on homometallic and heterometallic samples synthesized via chemical titration. The experimental patterns were compared to simulated patterns generated from the crystal structure of previously reported lanthanide-nitrate hexamers (ICSD #418810 for Eu; ICSD #418815 for Tb) with the formula, $[Ln_6(\mu_6\text{-}O)(\mu_3\text{-}OH)_8(NO_3)_6(H_2O)_{12}]\cdot2(NO_3)\cdot3(H_2O)$; Ln = Eu, Tb.[1] Samples synthesized via chemical (i.e. base) titration are in good agreement with the calculated patterns of with formation of the targeted phases (Figures S1 and S2).

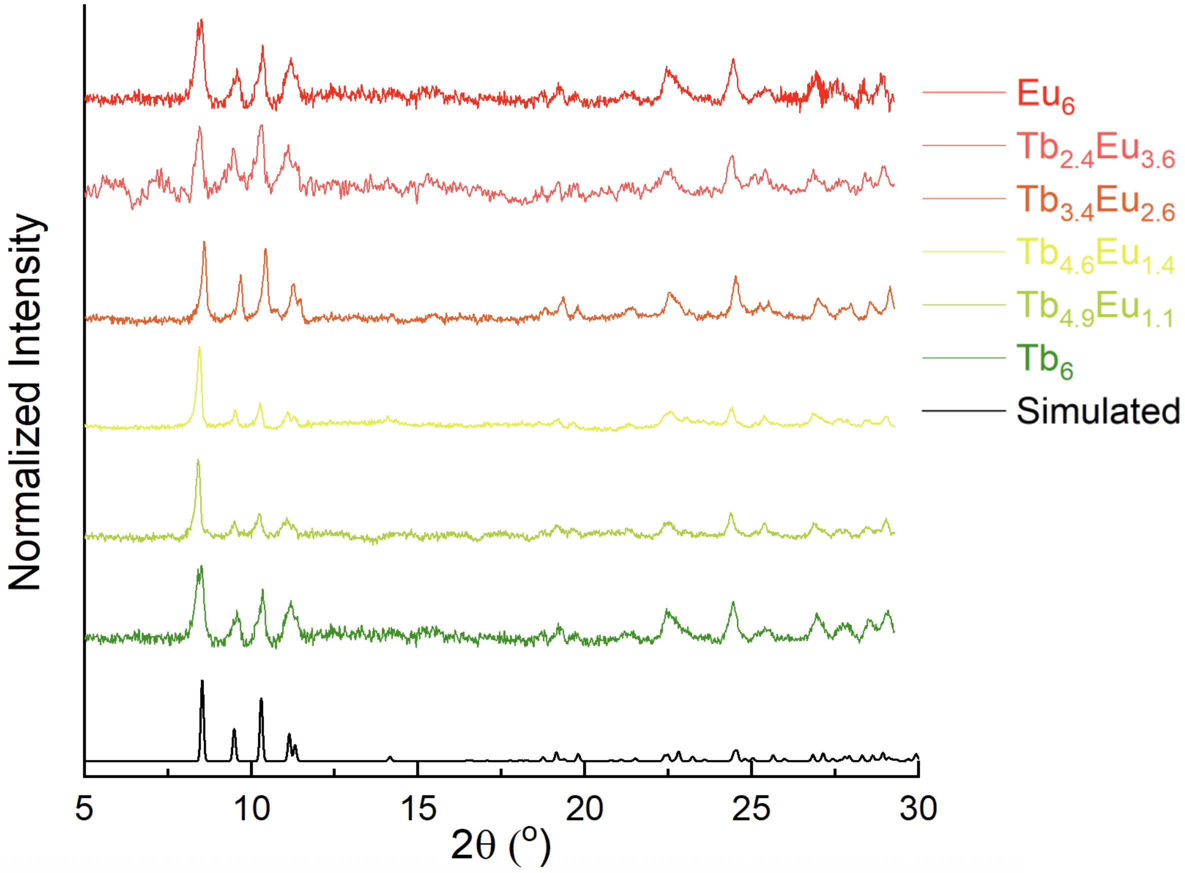

Figure S1: PXRD patterns of various $\mathbf{Tb_{6-x}Eu_x}$ mixed metal hexamers and the simulated PXRD pattern of $[Eu_6(\mu_6\text{-}O)(\mu_3\text{-}OH)_8(NO_3)_6(H_2O)_{12}]\cdot2(NO_3)\cdot3(H_2O)$ (black) (ICSD# 418810).

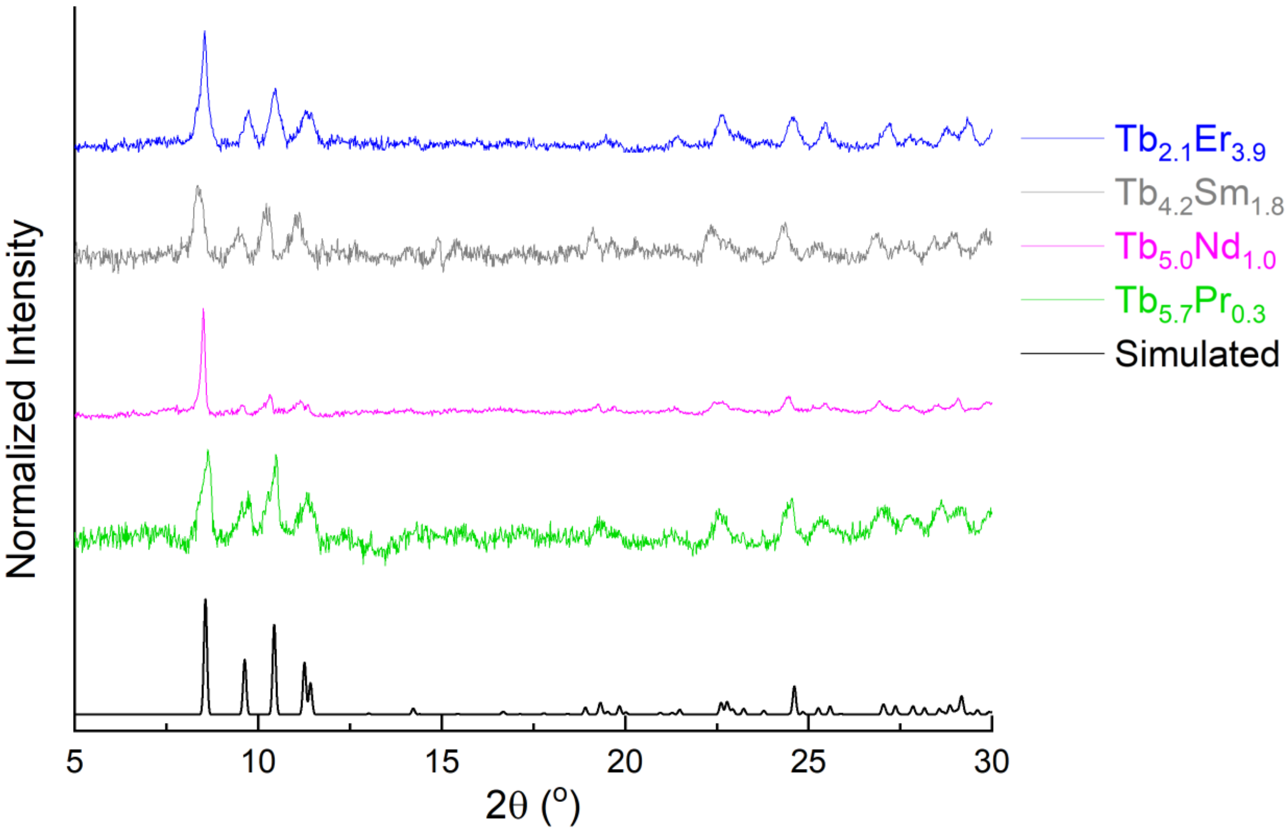

Figure S2: PXRD patterns of various $\mathbf{Tb_{6-x}Ln_x}$ mixed metal hexamers and the simulated PXRD pattern of $[Tb_6(\mu_6\text{-}O)(\mu_3\text{-}OH)_8(NO_3)_6(H_2O)_{12}]\bullet 2(NO_3)\bullet 3(H_2O)$ (black) (ICSD# 418815).

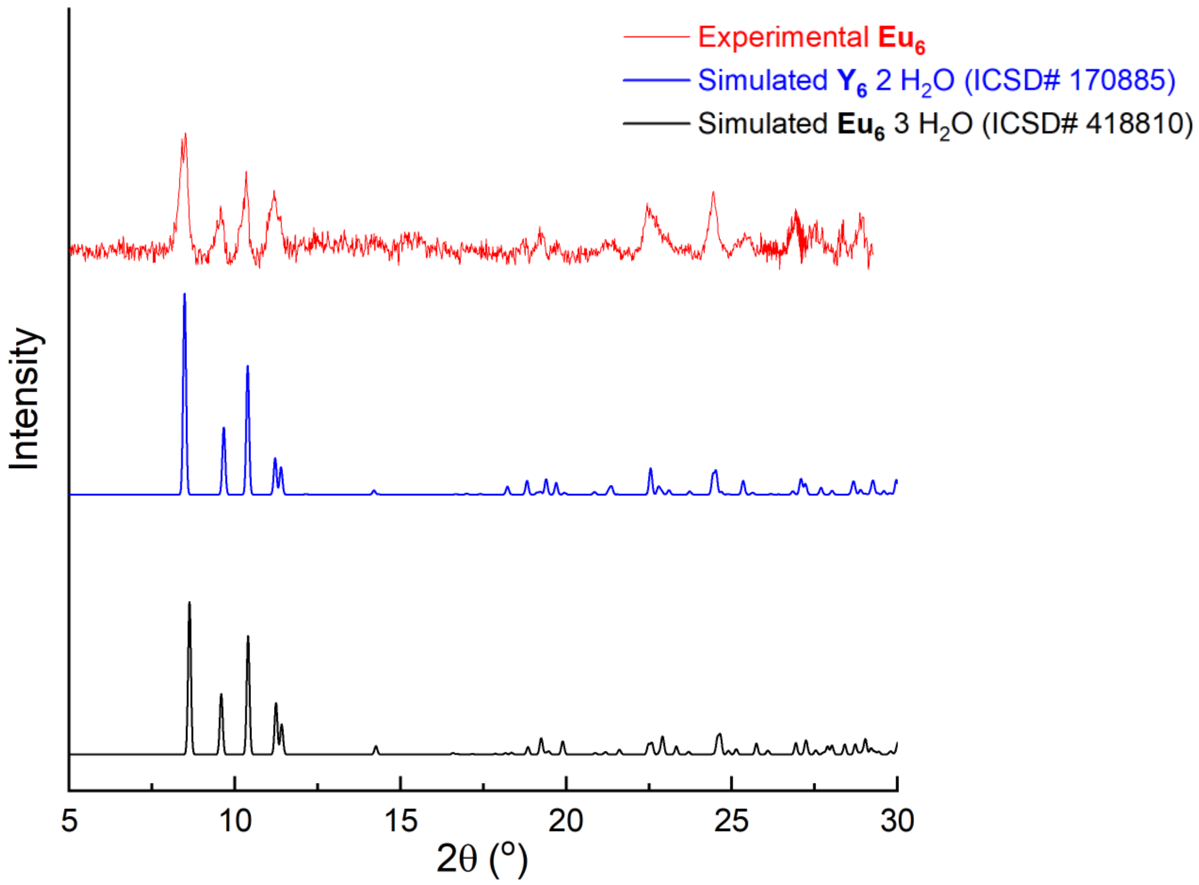

Figure S3: Experimentally obtained PXRD pattern of **Eu$_6$** (red) compared to the simulated pattern for **Y$_6$** (blue; ICSD # 170885) with two water molecules in the outer coordination sphere and the simulated pattern for **Eu$_6$** (black; ICSD #418810) with three water molecules in the outer coordination sphere. As only subtle differences are observed between the di- and tri-hydrate, we have formulated the compounds in the manuscript as $\cdot n H_2O$ reflecting uncertainty in the degree of hydration.

3. Nano-Electrospray Ionization-Mass Spectrometry

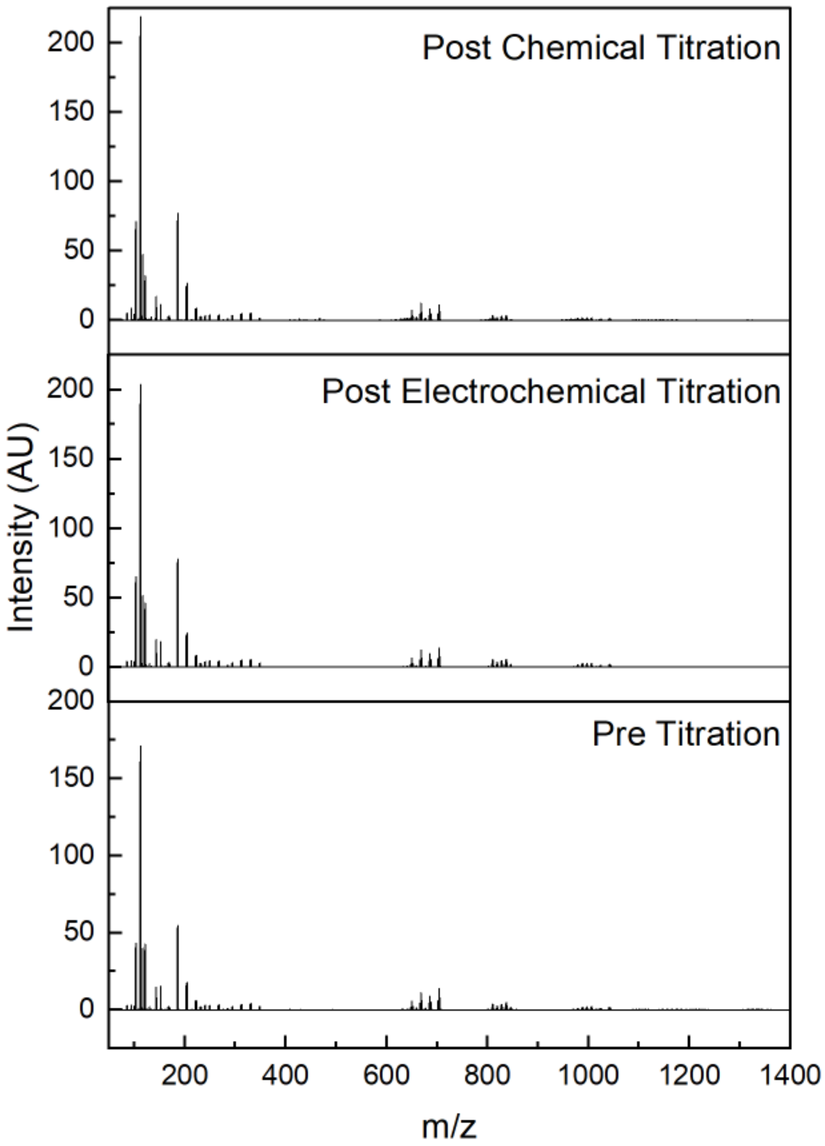

Figure S4: nESI-MS spectrum of pre-titration Eu nitrate solution (bottom), post-electrolysis Eu nitrate solution (middle), and post-chemical titration Eu nitrate solution (top).

Table S2: Experimental and simulated nESI-MS peaks with corresponding assigned species.

Experimental m/z	Simulated m/z	Assignment
85.47	85.4669	$Eu(H_2O)^{2+}$
94.48	94.4747	$Eu(H2O)_2{}^{2+}$
102.98	102.9748	$Eu(OH)(H_2O)_2{}^{2+}$
111.98	111.9781	$Eu(OH)(H_2O)_3{}^{2+}$
121.00	120.9897	$Eu(OH)(H_2O)_4{}^{2+}$
143.48	143.4805	$Eu(NO_3)(H_2O)_4{}^{2+}$
152.49	152.4863	$Eu(NO_3)(H_2O)_5{}^{2+}$
186.94	186.9371	$Eu(OH)_2{}^{+}$
204.95	204.9459	$Eu(OH)_2(H_2O)^{+}$
222.95	222.9546	$Eu(OH)_2(H_2O)_2{}^{+}$
231.92	231.9165	$Eu(OH)(NO_3)^{+}$
240.96	240.9649	$Eu(OH)_2(H_2O)_3{}^{+}$
249.93	249.932	$Eu(OH)(NO_3)(H_2O)^{+}$
267.94	267.9407	$Eu(OH)(NO_3)(H_2O)_2{}^{+}$
294.91	294.9108	$Eu(NO_3)_2(H_2O)^{+}$
312.93	312.9264	$Eu(NO_3)_2(H_2O)_2{}^{+}$
330.95	330.9476	$Eu(NO_3)_2(H_2O)_3{}^{+}$
348.95	348.9472	$Eu(NO_3)_2(H_2O)_4{}^{+}$
624.83	624.825	$Eu_4(NO_3)_{10}(H_2O)_3{}^{2+}$
651.82	651.8174	$Eu_2(NO_3)_5(H_2O)_2{}^{+}$
660.83	660.8264	$Eu_4(NO_3)_{10}(H_2O)_5{}^{2+}$
669.84	669.8393	$Eu_2(NO_3)_5(H_2O)_3{}^{+}$
678.83	678.8318	$Eu_4(NO_3)_{10}(H_2O)_7{}^{2+}$
687.84	687.8374	$Eu_2(NO_3)_5(H_2O)_4{}^{+}$
705.86	705.8604	$Eu_2(NO_3)_5(H2O)_5{}^{+}$
812.27	812.2701	$Eu_5(NO_3)_{13}(H_2O)_3{}^{2+}$
821.29	821.2858	$Eu_5(NO_3)_{13}(H_2O)_4{}^{2+}$
830.27	830.274	$Eu_5(NO_3)_{13}(H_2O)_5{}^{2+}$
839.30	839.2982	$Eu_5(NO_3)_{13}(H_2O)_6{}^{2+}$
848.29	848.293	$Eu_5(NO_3)_{13}(H_2O)_7{}^{2+}$
972.74	972.7357	$Eu_6(NO_3)_{16}(H_2O)_2{}^{2+}$
981.73	981.7313	$Eu_6(NO_3)_{16}(H_2O)_3{}^{2+}$
990.61	990.6135	$Eu_6(NO_3)_{16}(H_2O)_4{}^{2+}$
999.72	999.7194	$Eu_6(NO_3)_{16}(H_2O)_5{}^{2+}$
1008.71	1008.7108	$Eu_6(NO_3)_{16}(H_2O)_6{}^{2+}$
1017.73	1017.7283	$Eu_6(NO_3)_{16}(H_2O)_7{}^{2+}$
1026.74	1026.7429	$Eu_3(NO_3)_8(H_2O)_4{}^{+}$

4. Representative ICP-MS Data

Due to the large volume of ICP-MS data collected for this study, a representative dataset (**$Tb_{4.9}Eu_{1.1}$**) is presented below. All ICP-MS data collected for **$Tb_{6-x}Eu_x$** and the **$Tb_{6-x}Ln_x$** series were processed as described.

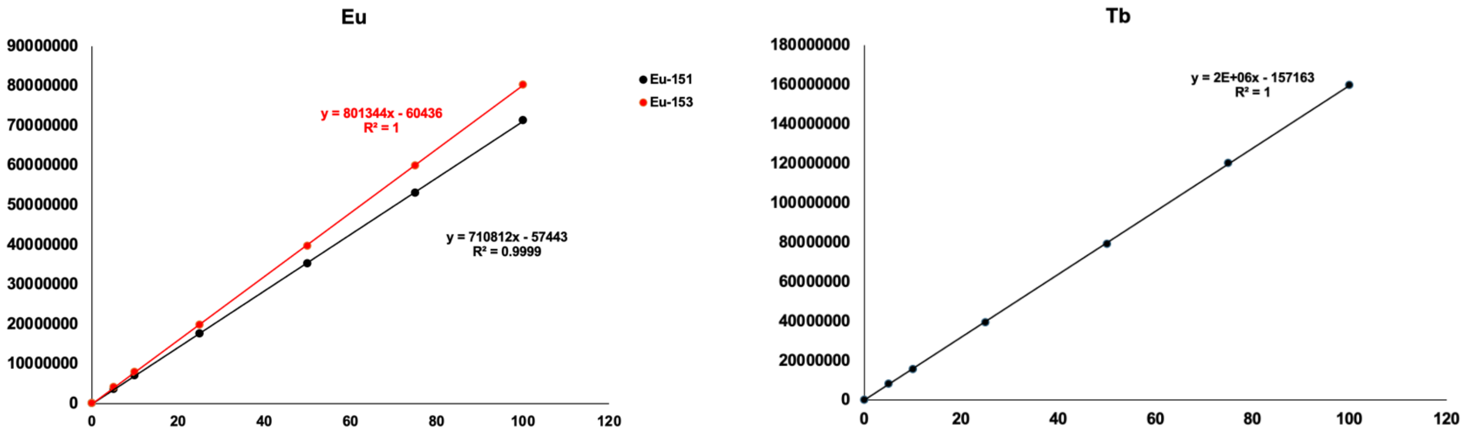

Figure S5: Calibration curves for europium and terbium samples used for trial 1 of **$Tb_{4.9}Eu_{1.1}$** ICP-MS data.

Table S3: Trial 1 data for **$Tb_{4.9}Eu_{1.1}$** ICP-MS data.

Trial 1				
Eu		151		153
2% HNO3	0	94918.37		108215.66
Eu 5ppb	5	3638619.49		4099168.25
Eu 10ppb	10	7018024.47		7913259.73
Eu 25ppb	25	17599563.57		19834625.05
Eu 50ppb	50	35252877.89		39694667.27
Eu 75ppb	75	53022260.64		59967620.01
Eu 100ppb	100	71336706.19		80315496.17
25% Eu	0.635921509	394577.64	0.631503399	445615.46
Tb		159		
2% HNO3	0	56695.41		
Tb 5ppb	5	8150614.83		
Tb 10ppb	10	15761446.33		

Tb 25ppb	25	39508439.8		
Tb 50ppb	50	79139894.61		
Tb 75ppb	75	120204017.3		
Tb 100ppb	100	159804267.6		
25% Eu	2.82301362	5488864.24		
Eu	Eu	Eu	Tb	Tb
conc. Ln (ppb)	151	0.635921509	conc. Tb (ppb)	2.82301362
conc. Ln (mol)	151	0.004211401	conc. Tb (mol)	0.017754803
ppb	153	0.631503399	mol %	0.809825242
mol	153	0.004127473		
ave		0.004169437		
mol %		0.190174758		

Table S4: Trial 2 data for $\mathbf{Tb_{4.9}Eu_{1.1}}$ ICP-MS data.

Trial 2				
Eu		151		153
2% HNO3	0	94918.37		108215.66
5ppb Eu	5	3638619.49		4099168.25
10ppb Eu	10	7018024.47		7913259.73
25ppb Eu	25	17599563.57		19834625.05
50ppb Eu	50	35252877.89		39694667.27
75ppb Eu	75	53022260.64		59967620.01
100ppb Eu	100	71336706.19		80315496.17
25% Eu	0.635921509	394577.64	0.631503399	445615.46
Tb		159		
2% HNO3	0	56695.41		
5ppb Tb	5	8150614.83		
10ppb Tb	10	15761446.33		
25ppb Tb	25	39508439.8		
50ppb Tb	50	79139894.61		
75ppb Tb	75	120204017.3		
100ppb Tb	100	159804267.6		
25% Eu	2.82301362	5488864.24		
Eu	Eu	Eu	Tb	Tb
conc. Ln (ppb)	151	0.635921509	conc. Tb (ppb)	2.82301362

conc. Ln (mol)	151	0.004211401	conc. Tb (mol)	0.017754803
ppb	153	0.631503399	mol %	0.809825242
mol	153	0.004127473		
ave		0.004169437		
mol %		0.190174758		

Table S5: Trial 3 data for $\mathbf{Tb_{4.9}Eu_{1.1}}$ ICP-MS data.

Trial 3				
Eu		151		
2% HNO_3	0	3196117.15		
5ppb Eu	5	3579552.73		
10ppb Eu	10	3826450.51		
25ppb Eu	25	5023704.88		
50ppb Eu	50	6837807.62		
75ppb Eu	75	9110917.37		
100ppb Eu	100	10631415.73		
25% Eu	13.65636923	4038266.44	5.927497214	
	159			
2% HNO_3	0	6943013.08		
5ppb Tb	5	9668970.61		
10ppb Tb	10	10602147.61		
25ppb Tb	25	13059988.59		
50ppb Tb	50	17322575.72		
75ppb Tb	75	22334898.9		
100ppb Tb	100	26747620.43		
25% Eu	62.4463392	19710561.99		
Eu	Eu	Eu	Tb	Tb
conc. Ln (ppb)	151	13.65636923	conc. Tb (ppb)	62.4463392
conc. Ln (mol)	151	0.090439531	conc. Tb (mol)	0.392744272
mol %		0.187174178	mol %	0.812825822

*Eu-153 measurements were found to be anomalously low for trial 3 and therefore only Eu-151 was considered

5. Separation Factors for $Tb_{6-x}Eu_x$

Table S6: $S_{Tb/Ln}$ calculated for the $\mathbf{Tb_{6-x}Eu_x}$.

Eu/Tb (mol%)	Separation Factor
25/75	1.4 ± 0.1
33/66	1.6 ± 0.2
57/43	1.6 ± 0.4
75/25	1.9 ± 0.4

Table S7: $S_{Tb/Ln}$ calculated for the $\mathbf{Tb_{6-x}Ln_x}$.

Ln	Separation Factor
Pr	23 ± 6
Nd	5.2 ± 0.3
Sm	2.3 ± 0.1
Er	0.6 ± 0.1

6. Luminescence Data of $Tb_{6-x}Eu_x$

Excitation and emission spectra were collected for **Eu_6** and **Tb_6**. Notably, the excitation spectra of both **Eu_6** (Figure S6) and **Tb_6** (Figure S7) feature a band at 320 nm which is attributed to a nitrate-ion absorption band.[13] All other transitions are characteristic of the metal centers.[14,15]

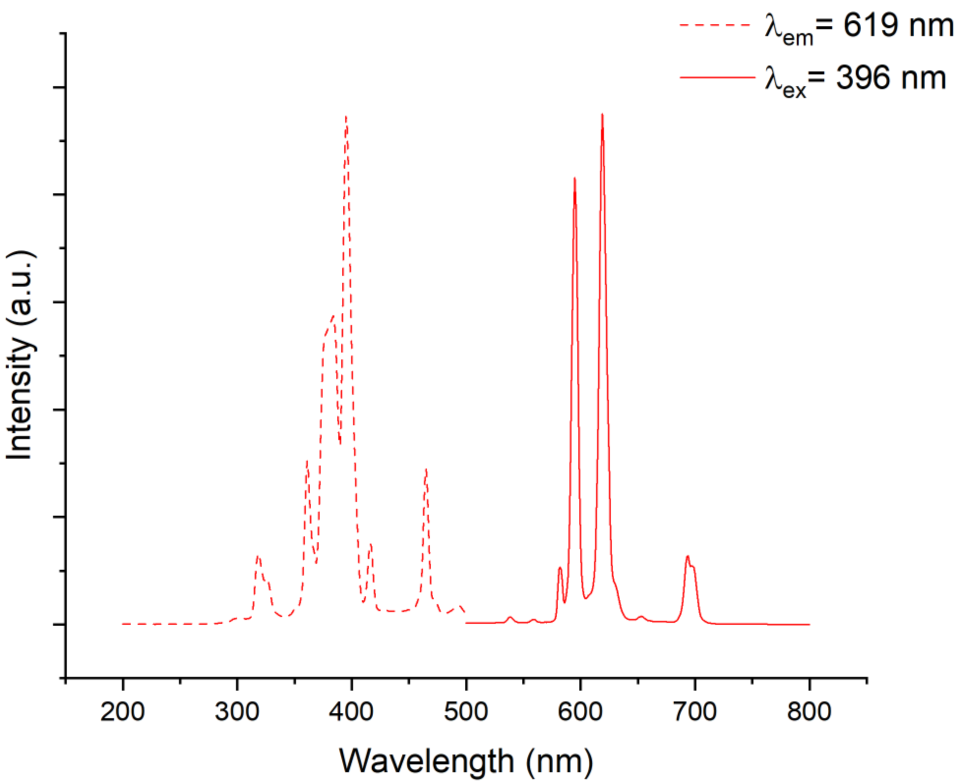

Figure S6: Excitation (dotted red line) and emission (solid red line) spectra of **Eu_6**.

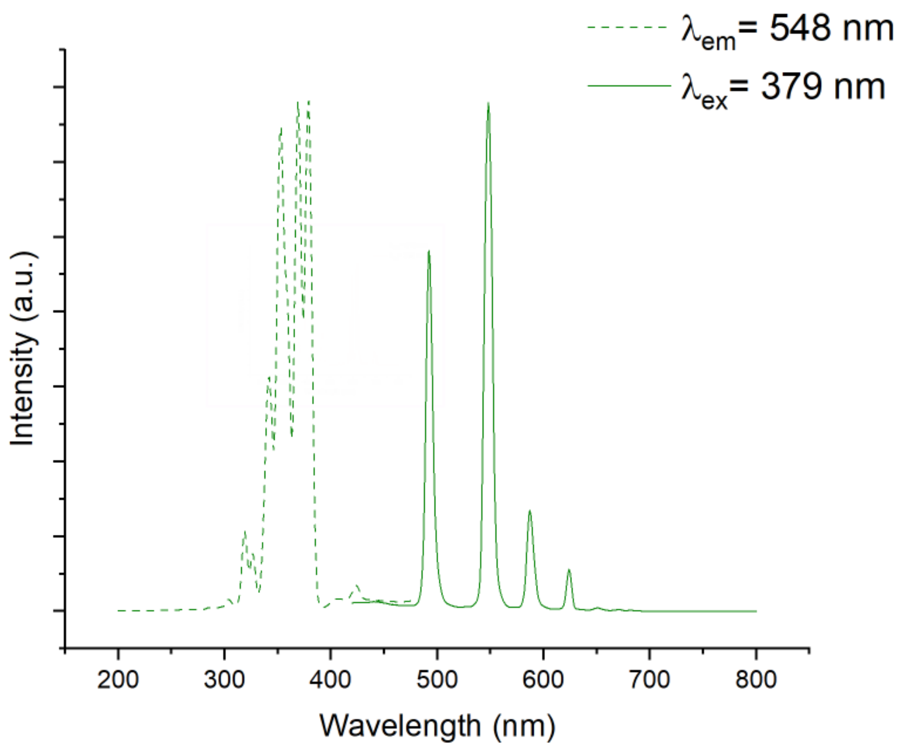

Figure S7: Excitation (dotted green line) and emission (solid green line) spectra of $\mathbf{Tb_6}$.

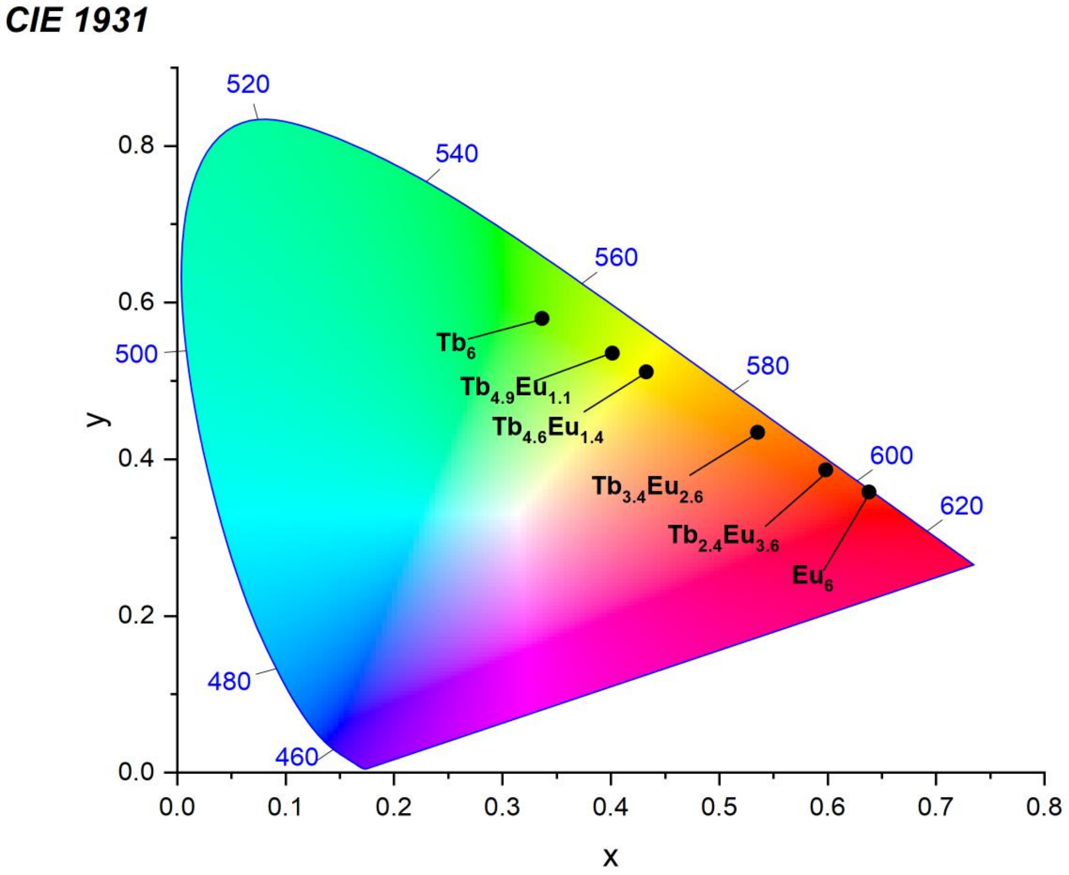

Figure S8: CIE plot for the $\mathbf{Tb_{6-x}Eu_x}$ series.

7. References

(1) Giester, G.; Žák, Z.; Unfried, P. Syntheses and Crystal Structures of Rare Earth Basic Nitrates Hydrates: Part III. $[Ln_6(\mu_6–O)(\mu_3–OH)_8(H_2O)_{12}(\eta_2–NO_3)_6](NO_3)_2 \cdot xH_2O$, Ln=Y, Sm, Eu, Gd, Tb, Dy, Ho, Er, Tm, Yb, Lu; x=3, 4, 5, 6. *Journal of Alloys and Compounds* **2009**, *481* (1), 116–128. https://doi.org/https://doi.org/10.1016/j.jallcom.2009.03.118.

(2) Mahé, N.; Guillou, O.; Daiguebonne, C.; Gérault, Y.; Caneschi, A.; Sangregorio, C.; Chane-Ching, J. Y.; Car, P. E.; Roisnel, T. Polynuclear Lanthanide Hydroxo Complexes: New Chemical Precursors for Coordination Polymers. *Inorganic Chemistry* **2005**, *44* (22), 7743–7750. https://doi.org/10.1021/ic0401086.

(3) Le Natur, F.; Calvez, G.; Daiguebonne, C.; Guillou, O.; Bernot, K.; Ledoux, J.; Le Pollès, L.; Roiland, C. Coordination Polymers Based on Heterohexanuclear Rare Earth Complexes: Toward Independent Luminescence Brightness and Color Tuning. *Inorganic Chemistry* **2013**, *52* (11), 6720–6730.

(4) Le Natur, F.; Calvez, G.; Guégan, J.-P.; Le Pollès, L.; Trivelli, X.; Bernot, K.; Daiguebonne, C.; Neaime, C.; Costuas, K.; Grasset, F.; Guillou, O. Characterization and Luminescence Properties of Lanthanide-Based Polynuclear Complexes Nanoaggregates. *Inorganic Chemistry* **2015**, *54* (12), 6043–6054. https://doi.org/10.1021/acs.inorgchem.5b00947.

(5) Calvez, G.; Guillou, O.; Daiguebonne, C.; Car, P.-E.; Guillerm, V.; Gérault, Y.; Le Dret, F.; Mahé, N. Octahedral Hexanuclear Complexes Involving Light Lanthanide Ions. *Inorganica Chimica Acta* **2008**, *361* (8), 2349–2356. https://doi.org/https://doi.org/10.1016/j.ica.2007.12.008.

(6) Yao, H.; Calvez, G.; Daiguebonne, C.; Bernot, K.; Suffren, Y.; Guillou, O. Hetero-Hexalanthanide Complexes: A New Synthetic Strategy for Molecular Thermometric Probes. *Inorganic Chemistry* **2019**, *58* (23), 16180–16193. https://doi.org/10.1021/acs.inorgchem.9b02668.

(7) Marsh, D. A.; Goberna-Ferrón, S.; Baumeister, M. K.; Zakharov, L. N.; Nyman, M.; Johnson, D. W. Ln Polyoxocations: Yttrium Oxide Solution Speciation & Solution Deposited Thin Films. *Dalton Transactions* **2017**, *46* (3), 947–955. https://doi.org/10.1039/C6DT03733J.

(8) Calvez, G.; Daiguebonne, C.; Guillou, O.; Pott, T.; Méléard, P.; Le Dret, F. Lanthanide-Based Hexanuclear Complexes Usable as Molecular Precursors for New Hybrid Materials. *Comptes Rendus Chimie* **2010**, *13* (6), 715–730. https://doi.org/https://doi.org/10.1016/j.crci.2010.03.029.

(9) Giester, G.; Unfried, P.; Žák, Z. Syntheses and Crystal Structures of Some New Rare Earth Basic Nitrates II:$[Ln_6O(OH)_8(H_2O)_{12}(NO_3)_6](NO_3)_2 \cdot xH_2O$, Ln= Sm, Dy, Er; x (Sm)= 6, x (Dy)= 5, x (Er)= 4. *Journal of Alloys and Compounds* **1997**, *257* (1–2), 175–181.

(10) Charushnikova, I. A.; Den Auwer, C. Crystal Structure of a New Basic Nitrate of Neodymium (III),$[Nd_6O(OH)_8(H_2O)_{14}(NO_3)_6](NO_3)_2 \cdot 2H_2O$. *Crystallography Reports* **2007**, *52*, 226–229.

(11) Raizada, M.; Shahid, M.; Hussain, S.; Ashafaq, M.; Siddiqi, Z. A. A New Antiferromagnetic Dy_6 Oxido-Material as a Multifunctional Aqueous Phase Sensor for Picric Acid as Well as Fe^{3+} Ions. *Materials Advances* **2020**, *1* (9), 3518–3531.

(12) Ẑák, Z.; Unfried, P.; Giester, G. The Structures of Some Rare Earth Basic Nitrates $[Ln_6(\mu_6\text{-}O)(\mu_3\text{-}OH)_8(H_2O)_{12}(NO_3)_6](NO_3)_2 \cdot xH_2O$ Ln= Y, Gd, Yb, x (Y, Yb)= 4; x (Gd)= 5. A Novel Rare Earth Metal Cluster of the M6X8 Type with Interstitial O Atom. *Journal of Alloys and Compounds* **1994**, *205* (1–2), 235–242.

(13) Blasse, G.; Dirksen, G. J.; van Vliet, J. P. M. The Luminescence of Europium Nitrate Hexahydrate, $Eu(NO_3)_3 \cdot 6H_2O$. *Inorganica Chimica Acta* **1988**, *142* (1), 165–168. https://doi.org/https://doi.org/10.1016/S0020-1693(00)80677-5.

(14) Medina-Velazquez, D. Y.; Caldiño, U.; Morales-Ramirez, A.; Reyes-Miranda, J.; Lopez, R. E.; Escudero, R.; Ruiz-Guerrero, R.; Perez, M. F. M. Synthesis of Luminescent Terbium-Thenoyltriflouroacetone MOF Nanorods for Green Laser Application. *Optical Materials* **2019**, *87*, 3–10.

(15) Bertry, L.; Durupthy, O.; Aschehoug, P.; Viana, B.; Chanéac, C. Experimental Evidence of Luminescence Quenching at Long Coupling Distances in Europium (III) Doped Core-Shell Gold Silica Nanoparticles. *Golden Bulletin* **2013**, *46*, 349–355.
